# Correction: Liu et al. *Epichloë gansuensis* Increases the Tolerance of *Achnatherum* *inebrians* to Low-P Stress by Modulating Amino Acids Metabolism and Phosphorus Utilization Efficiency. *J.* *Fungi* 2021, *7*, 390

**DOI:** 10.3390/jof7100813

**Published:** 2021-09-28

**Authors:** Yinglong Liu, Wenpeng Hou, Jie Jin, Michael J. Christensen, Lijun Gu, Chen Cheng, Jianfeng Wang

**Affiliations:** 1 State Key Laboratory of Grassland Agro-Ecosystems, Center for Grassland Microbiome, Key Laboratory of Grassland Livestock Industry Innovation, Ministry of Agriculture and Rural Affairs, Engineering Research Center of Grassland Industry, Ministry of Education, College of Pastoral Agriculture Science and Technology, Lanzhou University, Lanzhou 730000, China; liuyl2020@lzu.edu.cn (Y.L.); houwp19@lzu.edu.cn (W.H.); gulj@lzu.edu.cn (L.G.); chengch20@lzu.edu.cn (C.C.); 2 Key Laboratory of Cell Activities and Stress Adaptations, Ministry of Education, School of Life Sciences, Lanzhou University, Lanzhou 730000, China; jinj2015@lzu.edu.cn; 3 Retired Scientist of AgResearch, Grasslands Research Centre, Private Bag 11-008, Palmerston North 4442, New Zealand; mchristensenpn4410@gmail.com

**Error in Figure**

In the original article [1], the wrong “Figure 2a,b” were uploaded due to author’s negligence, there were mistakes in “Figure 2a,b” as published. The corrected “Figure 2a,b” appears below, and the corresponding detailed content have also been corrected. The authors apologize for any inconvenience caused and state that the scientific conclusions are unaffected. The original article has been updated.

**Text Correction**

In the original article, Abstract: “Further, *Epichloë gansuensis* increased C content of roots compared to the root of E− plant at 0.01 mM P and 0.5 mM P” was not described correctly, should be changed to: “Further, low P stress increased C content of leaves of E+ *Achnatherum inebrians* compared to 0.5 mM P”.

In the original article, the Section 3.2 of Result: “We found that *Epichloë gansuensis* significantly increased the C content of roots at 0.01 and 0.5 mM P concentrations, and the C content of E+ roots at 0.01 and 0.5 mM P concentration was 1.55 and 1.14 times higher than that of E−, respectively (Figure 2d).” was not described correctly, should be changed to: “We found that low P stress significantly increased the leaves C content of E+ *Achnatherum inebrians* compared with 0.5 mM P, and the C content of E+ leaves at 0.01 mM P was 1.11 times higher than that at 0.5 mM P (Figure 2a). However, the presence of *Epichloë gansuensis* did not significantly affect the C content of leaves and roots at 0.01 and 0.5 mM P, respectively, (Figure 2a,b).”

In the original article, the Section 4 of Discussion: “However, low P had no significant effect on C content in leaves and roots of E+ and E− plants, which may be due to that the low P stress has limited C uptake by *Epichloë gansuensis* and plants. Interestingly, *Epichloë gansuensis* increased the content of C in roots, but had no significant effect on leaves, which implied that *Epichloë gansuensis* may control the transfer of host C nutrients.” was not described correctly, should be changed to: “*Epichloë gansuensis* did not significantly affect the C content of leaves and roots at 0.01 and 0.5 mM P, respectively. However, 0.01 mM P increased the C content of leaves of E+ plant compared to 0.5 mM P.”

In the original article, the Section 4 of Discussion: “This is similar to a previous study that suggested that the effect of endophyte on the nutrient content of plant roots is greater than that of aboveground parts [32]” was not described correctly, should be changed to: “The previous study showed that the effect of mycorrhizal fungus on the nutrient content of plant roots is greater than that of aboveground parts [32], which was not consistent with our results, perhaps *Epichloë gansuensis* only exist in plant tissues other than roots, so low P stress increased the C content of leaves of E+ plants.”

In the original article, the Section 4 of Discussion: “In addition, other studies have reported that the increase of plant nutrient uptake is related to the increase of root surface area. Perhaps *Epichloë gansuensis* enhances root exudation and microbial activity, thus leading to more nutrients for plant roots [34].” was not described correctly, should be changed to: “One study showed that rhizobacteria enhance root exudation and microbial activity, thus leading to more nutrients for plant roots [34].”

## Figures and Tables

**Figure 2:**
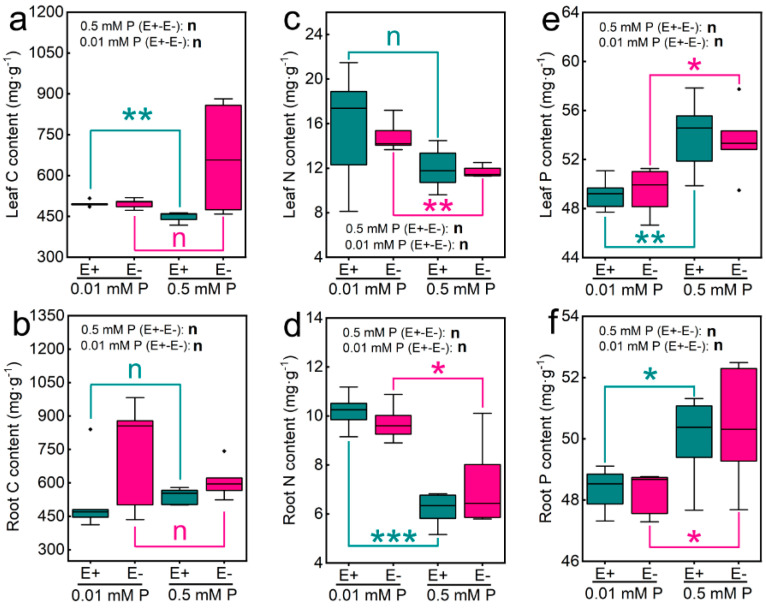
The content of C, N, and P of leaves (**a**,**c**,**e**) and roots (**b**,**d**,**f**) in E− and E+ plants at 0.01 and 0.5 mM P. *, ** and *** showed differences at *p* < 0.05, *p* < 0.01, and *p* < 0.001, respectively.
