# Supplementary material for: Epichloë gansuensis Increases the Tolerance of Achnatherum inebrians to Low-P Stress by Modulating Amino Acids Metabolism and Phosphorus Utilization Efficiency
Source: J Fungi (Basel). 2021 May 17;7(5):390. doi: 10.3390/jof7050390 (PMC8156409; doi:10.3390/jof7050390)
Supplement: Supplementary file 1 [file jof-07-00390-s001.zip › jof-1215289-supplementary/Supplementary Information-Table 1.pdf]

Table S1. Relative concentrations and fold-changes in the levels of significantly different metabolites in leaves of E+ and E- *A. inebrians* seedlings under low phosphorus stress. Fold changes were calculated using the formula $Log_2^{(LE+/LE-)}$ and $Log_2^{(0.01\ mM/0.5\ mM)}$. *, **; *** mean significant differences at $P < 0.05$; $P < 0.01$ and $P < 0.001$, respectively.

**Metabolites name**	**0.01 mM**		
	Relative concentration		Fold Changes
	LE+	LE-	$Log_2^{(LE+/LE-)}$
Histidine	0.001 ± 0.00	121.38 ± 30.33	-16.42 **
Lactulose	38.32 ± 38.31	799.22 ± 310.66	-4.38 *
5-Aminovaleric acid	268.10 ± 30.01	72.60 ± 46.06	1.88 **
Vanillin	197.55 ± 53.77	33.48 ± 33.48	2.56 *
Citric acid	26664.74 ± 12043.71	54918.77 ± 3620.41	-1.04 *
1-Kestose	1.56 ± 1.56	17.25 ± 6.73	-3.46 *
Sedoheptulose	243.36 ± 70.39	1284.66 ± 124.17	-2.40 ***
4-hydroxycinnamic acid	145.39 ± 55.29	781.32 ± 78.71	-2.43 ***
D-Glutamic acid	630.43 ± 38.80	199.45 ± 59.48	1.66 ***
1-Hexadecanol	94.46 ± 31.88	312.88 ± 36.80	-1.73 **
Ascorbate	74.55 ± 28.67	255.25 ± 36.88	-1.78 **
Pyruvic acid	228.19 ± 53.15	379.42 ± 29.09	-0.73 *
4-Acetamidobutyric acid	11011.04 ± 982.49	3786.65 ± 1175.53	1.54 ***
	**0.5 mM**		
	Relative concentration		Fold Changes
	LE+	LE-	$Log_2^{(LE+/LE-)}$
Benzoic acid	21.23 ± 21.23	118.63 ± 9.68	-2.48 **
Citramalic acid	136.63 ± 8.54	75.57 ± 24.25	0.85 *
Cortexolone	573.28 ± 172.47	3346.60 ± 732.10	-2.55 **
Salicylic acid	577.95 ± 162.73	203.98 ± 40.80	1.50 *
	**LE+**		
	Relative concentration		Fold Changes
	0.01 mM	0.5 mM	$Log_2^{(0.01\ mM/0.5\ mM)}$
Lysine	6262.47 ± 965.22	0.002 ± 0.00	21.75 ***
Glutamine	4834.62 ± 1642.77	0.002 ± 0.00	21.37 *
Biuret	825.17 ± 60.91	0.002 ± 0.00	18.82 ***
D-Glutamic acid	630.43 ± 38.80	0.002 ± 0.00	18.44 ***
Asparagine	47899.74 ± 5352.97	4.70 ± 3.22	13.32 ***
Noradrenaline	217.28 ± 20.63	0.002 ± 0.00	16.90 ***
Orotic acid	98.08 ± 14.36	0.002 ± 0.00	15.75 ***

O-acetylserine	25.41 ± 1.80	0.002 ± 0.00	13.80 ***
Saccharic acid	662.07 ± 211.23	95.54 ± 95.54	2.79 *
Vanillin	197.55 ± 53.77	0.002 ± 0.00	16.76 **
Benzoic acid	110.64 ± 6.63	21.232 ± 21.23	2.38 **
Urea	471.77 ± 37.56	1.45 ± 0.78	8.34 ***
Glucose-6-phosphate	8.15 ± 5.88	346.07 ± 144.65	-5.41 *
3-Cyano-L-alanine	20497.05 ± 2139.44	41.64 ± 17.16	8.94 ***
Glycocyamine	67.08 ± 14.47	7.69 ± 4.86	3.12 **
Taxifolin	153.65 ± 50.15	0.002 ± 0.00	16.40 *
Citric acid	26664.74 ± 12043.71	63433.32 ± 3990.86	-1.25 *
Alpha-ketoisocaproic acid	39.27 ± 12.55	0.002 ± 0.00	14.43 *
Xylitol	33.20 ± 3.47	7.871 ± 5.01	2.08 **
4-Methylcatechol	10.47 ± 3.51	0.002 ± 0.00	12.52 *
Thymol	88.21 ± 4.23	21.84 ± 10.95	2.01 ***
Tyrosine	6016.24 ± 1919.57	148.33 ± 85.20	5.34 *
Proline	12930.79 ± 1397.58	404.89 ± 173.63	5.00 ***
1-Kestose	1.56 ± 1.56	79.87 ± 26.67	-5.68 *
Thymidine	142.18 ± 26.82	15.77 ± 5.19	3.17 ***
Indole-3-acetamide	28.36 ± 9.44	0.74 ± 0.74	5.26 *
Isoleucine	14490.68 ± 1313.03	1174.12 ± 177.00	3.63 ***
Phosphomycin	39.04 ± 13.03	140.65 ± 10.26	-1.85 ***
Putrescine	10388.99 ± 687.66	1194.62 ± 303.56	3.12 ***
Itaconic acid	641.23 ± 84.83	319.49 ± 102.13	1.01 *
Phenylalanine	6424.97 ± 651.36	793.07 ± 131.54	3.02 ***

	**LE-**		
	Relative concentration		Fold Changes
	0.01 mM	0.5 mM	$Log_2^{(0.01\ mM/0.5\ mM)}$
Lysine	4992.98 ± 740.86	0.002 ± 0.00	21.51 ***
Asparagine	27917.25 ± 4577.11	0.87 ± 0.87	14.96 ***
Glutamine	3580.71 ± 1058.28	0.002 ± 0.00	21.03 **
4-Acetamidobutyric acid	3786.65 ± 1175.53	0.002 ± 0.00	21.11 **
Biuret	693.46 ± 42.98	0.002 ± 0.00	18.67 ***
Noradrenaline	275.88 ± 40.09	0.002 ± 0.00	17.33 ***
3-Cyano-L-alanine	8765.00 ± 1301.83	16.976 ± 11.24	9.01 ***
Alpha-Tocopherol	60.85 ± 60.85	670.59 ± 139.77	-3.46 **
Histidine	121.38 ± 30.33	0.002 ± 0.00	16.15 **
D-Glutamic acid	199.45 ± 59.48	0.002 ± 0.00	16.86 **
1-Aminocyclopropanecarboxylic acid	11.08 ± 11.08	116.71 ± 5.80	-3.40 ***

Urea	260.75 ± 40.48	1.42 ± 1.02	7.52 ***
Thymidine	181.85 ± 38.50	8.40 ± 5.31	4.43 **
Glucose-6-phosphate	14.80 ± 9.36	285.33 ± 110.87	-4.27 *
Putrescine	7328.38 ± 783.06	458.24 ± 205.62	4.00 ***
Oxalacetic acid	171.73 ± 29.17	32.93 ± 25.65	2.38 **
Tyrosine	11919.11 ± 1530.37	134.28 ± 76.86	6.47 ***
Orotic acid	62.77 ± 13.55	7.14 ± 7.14	3.14 **
O-acetylserine	24.77 ± 8.05	0.002 ± 0.00	13.86 *
Itaconic acid	528.91 ± 44.95	126.582 ± 67.51	2.06 ***
Gentisic acid	25.397 ± 16.16	198.52 ± 46.71	-2.97 **
Glycocyamine	50.19 ± 16.76	3.46 ± 3.46	3.86 *
Pelargonic acid	30.14 ± 19.07	170.60 ± 54.57	-2.51 *
Proline	7005.05 ± 1292.10	347.90 ± 97.31	4.33 ***
6-phosphogluconic acid	24.16 ± 8.77	218.495 ± 42.03	-3.18 **
Phthalic acid	112.50 ± 32.27	0.522 ± 0.26	7.75 **
Indole-3-acetamide	30.26 ± 11.07	3.509 ± 3.507	3.11 *
Cholic acid	889.32 ± 80.68	66.33 ± 21.02	3.75 ***
Maltotriose	51.42 ± 17.37	275.82 ± 83.84	-2.42 *
Pantothenic acid	311.67 ± 74.60	64.32 ± 53.00	2.28 *
Isoleucine	13103.16 ± 3297.08	1296.15 ± 102.21	3.34 *
Phenylalanine	5751.03 ± 367.50	862.41 ± 65.19	2.74 ***
