# Supplementary material for: Epichloë gansuensis Increases the Tolerance of Achnatherum inebrians to Low-P Stress by Modulating Amino Acids Metabolism and Phosphorus Utilization Efficiency
Source: J Fungi (Basel). 2021 May 17;7(5):390. doi: 10.3390/jof7050390 (PMC8156409; doi:10.3390/jof7050390)
Supplement: Supplementary file 1 [file jof-07-00390-s001.zip › jof-1215289-supplementary/Supplementary Information-Table 2.pdf]

**Table S2.** Relative concentrations and fold changes in the levels of significantly different metabolites in roots of E+ and E- *A.inebrians* seedlings under low phosphorus stress. Fold changes were calculated using the formula $Log_2^{(RE+/RE-)}$ and $log_2^{(0.01\ mM/0.5\ mM)}$. *, **, *** mean significant differences at *P*<0.05; *P*<0.01 and *P*<0.001, respectively.

| **Metabolites name** | **0.01 mM** | | |
|---|---|---|---|
| | Relative concentration | | Fold Changes |
| | RE+ | RE- | $Log_2^{(RE+/RE-)}$ |
| Cis-1,2-Dihydronaphthalene-1,2-diol | 112.71 ± 10.12 | 6.47 ± 6.47 | 4.12 *** |
| Ribonic acid | 21.78 ± 21.78 | 131.18 ± 14.33 | -2.59 ** |
| Tryptophan | 22.53 ± 14.32 | 101.37 ± 22.04 | -2.17 * |
| Maleic acid | 4353.06 ± 1517.48 | 8635.84 ± 658.09 | -0.99 * |
| Gluconic acid | 1088.21 ± 242.66 | 193.55 ± 9.59 | 2.49 ** |
| Cortexolone | 43.87 ± 9.90 | 105.88 ± 10.65 | -1.27 ** |
| 5-Aminovaleric acid | 283.62 ± 65.95 | 515.30 ± 18.51 | -0.86 ** |
| Proline | 5621.40 ± 462.78 | 13221.57 ± 1234.01 | -1.23 *** |
| Acetol | 46.71 ± 10.16 | 80.47 ± 4.64 | -0.78 * |
| Leucine | 629.65 ± 66.17 | 1447.81 ± 125.26 | -1.20 *** |
| 3-Methylglutaric acid | 49.03 ± 6.98 | 27.03 ± 6.48 | 0.86 * |
| Maleamate | 1284.51 ± 100.78 | 2466.03 ± 203.94 | -0.94 *** |
| Asparagine | 31739.20 ± 1973.62 | 16926.31 ± 1390.00 | 0.91 *** |
| D-Glutamic acid | 436.92 ± 27.61 | 231.55 ± 32.49 | 0.92 *** |
| Ethanolamine | 8764.70 ± 985.01 | 15408.24 ± 1246.48 | -0.81 ** |
| Alanine | 78297.52 ± 7430.11 | 130879.38 ± 7682.73 | -0.74 *** |
| 3-Cyano-L-alanine | 65566.20 ± 3491.10 | 38971.36 ± 4572.38 | 0.75 *** |
| | **0.5 mM** | | |
| | Relative concentration | | Fold Changes |
| | RE+ | RE- | $Log_2^{(RE+/RE-)}$ |
| Galactonic acid | 6.16 ± 2.57 | 0.003 ± 0.00 | 11.13 * |
| 1-Kestose | 382.755 ± 81.513 | 61.99 ± 25.93 | 2.63 ** |
| Uridine | 90.31 ± 27.78 | 3.18 ± 0.69 | 4.83 * |
| Isomaltose | 238.96 ± 78.48 | 39.64 ± 17.92 | 2.59 * |
| Asparagine | 292.53 ± 33.49 | 50.32 ± 16.57 | 2.54 *** |
| Cis-gondoic acid | 25.65 ± 2.76 | 6.78 ± 2.11 | 1.92 *** |
| Phosphate | 1007.46 ± 40.52 | 392.66 ± 159.62 | 1.36 ** |
| 3-hydroxybutyric acid | 223.03 ± 32.04 | 79.16 ± 22.57 | 1.49 ** |
| 3-Cyano-L-alanine | 1616.88 ± 168.88 | 589.59 ± 39.25 | 1.46 *** |
| Proline | 3000.47 ± 285.41 | 1480.16 ± 171.72 | 1.02 ** |

| | | | |
|---|---|---|---|
| 1-Hexadecanol | 832.73 ± 81.76 | 421.33 ± 74.21 | 0.98 ** |
| 1,3-diaminopropane | 104.71 ± 12.52 | 219.72 ± 33.12 | -1.07 ** |
| Aspartic acid | 5370.21 ± 434.28 | 2837.17 ± 375.08 | 0.92 ** |
| Alanine | 28742.71 ± 2411.05 | 17560.24 ± 2302.60 | 0.71 ** |
| Gluconic acid | 749.07 ± 124.84 | 418.33 ± 51.65 | 0.84 * |
| Tyrosine | 1229.14 ± 70.13 | 761.89 ± 143.81 | 0.69 * |
| Maltotriose | 642.36 ± 79.38 | 385.61 ± 40.00 | 0.74 * |
| Glutamic acid | 774.00 ± 44.31 | 499.91 ± 47.02 | 0.63 ** |
| | **RE+** | | |
| | Relative concentration | | Fold Changes |
| | 0.01 mM | 0.5 mM | $Log_2^{(0.01\ mM/0.5\ mM)}$ |
| Glutamine | 3133.63 ± 668.78 | 0.003 ± 0.00 | 20.13 *** |
| D-Glutamic acid | 436.92 ± 27.61 | 0.003 ± 0.00 | 17.28 *** |
| Benzoic acid | 133.22 ± 32.44 | 0.003 ± 0.00 | 15.57 ** |
| 6-phosphogluconic acid | 2.04 ± 2.04 | 39.99 ± 5.03 | -4.29 *** |
| Urea | 96.67 ± 19.76 | 0.49 ± 0.24 | 7.62 *** |
| Biuret | 1981.33 ± 117.45 | 90.51 ± 40.96 | 4.45 *** |
| 1-Aminocyclopropanecarboxylic acid | 34.45 ± 21.89 | 167.45 ± 10.04 | -2.28 *** |
| Lysine | 6726.81 ± 454.80 | 534.10 ± 260.50 | 3.65 *** |
| Glutaric Acid | 56.14 ± 35.57 | 222.33 ± 24.15 | -1.99 ** |
| Asparagine | 71188.01 ± 3123.29 | 292.53 ± 33.49 | 7.93 *** |
| Phthalic acid | 259.51 ± 11.93 | 2.72 ± 1.09 | 6.58 *** |
| Glucose-6-phosphate | 0.002 ± 0.00 | 24.15 ± 8.07 | -13.66 * |
| Phosphate | 354.30 ± 161.22 | 1007.42 ± 40.52 | -1.51 ** |
| 3-Cyano-L-alanine | 65566.19 ± 3491.10 | 1616.88 ± 168.88 | 5.34 *** |
| 1-Kestose | 40.96 ± 16.64 | 382.76 ± 81.51 | -3.22 ** |
| Uridine | 3.14 ± 1.25 | 90.31 ± 27.78 | -4.84 * |
| Ribitol | 1857.94 ± 270.77 | 652.15 ± 219.71 | 1.51 ** |
| Phosphomycin | 46.36 ± 15.32 | 169.94 ± 8.07 | -1.87 *** |
| Lauric acid | 123.70 ± 47.07 | 285.588 ± 25.16 | -1.21 * |
| Threonine | 46.24 ± 9.86 | 362.32 ± 8.57 | -2.97 *** |
| Aspartic acid | 35567.04 ± 1886.73 | 5370.21 ± 434.28 | 2.73 *** |
| L-Allothreonine | 16238.23 ± 822.45 | 3465.59 ± 192.14 | 2.23 *** |
| Fructose | 146912.22 ± 29603.53 | 373164.16 ± 8045.34 | -1.34 *** |
| Glycine | 10286.17 ± 1306.20 | 2531.83 ± 336.47 | 2.02 *** |
| | **RE-** | | |
| | Relative concentration | | Fold Changes |
| | 0.01 mM | 0.5 mM | $Log_2^{(0.01\ mM/0.5\ mM)}$ |
| Glutamine | 5384.25 ± 2044.41 | 0.003 ± 0.00 | 20.9 * |

| | | | |
|---|---|---|---|
| Biuret | 2104.12 ± 119.60 | 0.003 ± 0.00 | 19.55 *** |
| D-Glutamic acid | 231.55 ± 32.49 | 0.003 ± 0.00 | 16.36 *** |
| Cis-1,2-Dihydronaphthalene-1,2-diol | 6.47 ± 6.47 | 154.30 ± 24.40 | -4.58 *** |
| Lysine | 5439.91 ± 926.36 | 159.82 ± 101.11 | 5.09 *** |
| Benzoic acid | 161.76 ± 43.64 | 28.32 ± 28.32 | 2.51 * |
| 6-phosphogluconic acid | 0.002 ± 0.00 | 36.32 ± 8.92 | -14.48 ** |
| Gluconic lactone | 64.94 ± 16.98 | 0.003 ± 0.00 | 14.53 ** |
| Asparagine | 43121.44 ± 3062.46 | 50.32 ± 16.57 | 9.74 *** |
| Glycocyamine | 26.24 ± 6.39 | 0.003 ± 0.00 | 13.22 ** |
| Urea | 83.27 ± 9.27 | 2.04 ± 0.99 | 5.35 *** |
| Maleic acid | 8635.84 ± 658.09 | 1625.53 ± 727.88 | 2.41 *** |
| Ribitol | 1980.23 ± 267.28 | 481.92 ± 219.46 | 2.04 ** |
| 3-Cyano-L-alanine | 38971.36 ± 4572.38 | 589.59 ± 39.25 | 6.05 *** |
| Fructose | 123659.34 ± 39542.13 | 445575.80 ± 16662.40 | -1.85 *** |
| Phthalic acid | 127.35 ± 8.17 | 4.70 ± 0.74 | 4.76 *** |
| Tryptophan | 101.37 ± 22.04 | 23.89 ± 16.62 | 2.09 * |
| Lauric acid | 83.11 ± 30.76 | 369.28 ± 35.90 | -2.15 *** |
| Threonine | 25.99 ± 9.98 | 346.77 ± 67.16 | -3.74 *** |
| Aspartic acid | 31046.49 ± 1476.09 | 2837.17 ± 375.08 | 3.45 *** |
| Glutaric Acid | 86.39 ± 28.45 | 244.54 ± 25.31 | -1.5 ** |
| Proline | 13221.57 ± 1234.00 | 1480.16 ± 171.72 | 3.16 *** |
| Alanine | 130879.38 ± 7682.73 | 17560.24 ± 2302.60 | 2.9 *** |
| Glycine | 14128.19 ± 1138.62 | 2004.19 ± 164.70 | 2.82 *** |
| Threitol | 25.90 ± 8.55 | 61.82 ± 5.52 | -1.26 ** |
| Oxoproline | 99455.60 ± 6816.90 | 18089.21 ± 2078.31 | 2.46 *** |
| L-Allothreonine | 12947.93 ± 917.83 | 2471.45 ± 265.55 | 2.39 *** |
| Maleamate | 2466.03 ± 203.94 | 491.438 ± 101.32 | 2.33 *** |
| Serine | 27155.45 ± 1498.46 | 5632.65 ± 545.42 | 2.27 *** |
| Glutamic acid | 2246.39 ± 97.56 | 499.91 ± 47.02 | 2.17 *** |
| Thymidine | 95.15 ± 6.15 | 27.81 ± 5.61 | 1.77 *** |
